# Virtually learning as we go: Reflections on medical education through COVID-19

**DOI:** 10.36834/cmej.70531

**Published:** 2020-12-07

**Authors:** Kerry Boyd, Mohammad S. Zubairi

**Affiliations:** 1 Division of Child and Adolescent Psychiatry, Department of Psychiatry & Behavioural Neurosciences, McMaster University, Ontario, Canada; 2 Division of Developmental Paediatrics, Department of Pediatrics, McMaster University, Ontario, Canada; 3 McMaster Education Research, Innovation and Theory (MERIT) Program, McMaster University, Ontario, Canada

For over three months now, we have worked virtually from home, connecting with patients and their families through video conferencing or other digital technologies. Between us, we represent the fields of Psychiatry and Developmental Pediatrics. We frequently have physician trainees in child development and mental health join us in clinics. As clinicians, educators, and perpetual learners, we are accustomed to reflecting on ways to blend care and training needs. With pandemic restrictions for practice and educational settings, we are compelled to work, teach, and learn virtually. We are virtually learning as we go.

As clinicians, we are working from home. This is truly a privilege. We recognize unforeseen efficiencies (e.g. no traffic) and inefficiencies (e.g. bandwidth). We experience benefits of being home and side effects of work from home without previous set boundaries. We have a new appreciation for leadership and teamwork required to enable remote clinical care where possible. We know there are many more who are facing challenges in isolation.

As educators, we are also adapting to an evolving learning landscape. Remote records and virtual meetings are the portal for gaining clinical experience. At times it feels like a tenuous and anxious dependency on technology. Collaborative problem solving and persistence have been absolute necessities as we troubleshoot technical barriers to connecting with families. Virtual meetings have provided opportunities to directly observe residents as they interview families and receive feedback on their interaction. Residents do not need to repeat and ‘present’ a patient’s history in or out of the virtual clinic room. Patients and families who can share their experiences continue to prepare trainees for future practice.

As perpetual learners, continuing medical education is an even more pressing concern. We are virtually inundated with COVID-19 updates and virtual learning opportunities. “Spam” cannot filter for us the plethora of enticing webinars and workshops from narrative medicine to modifying clinical assessment. We are in the process of rethinking what, when and how best to learn. We are virtually learning about virtual learning.

The virtual portals into the lives of families, albeit limited, are opening up new ways to learn about needs. We are learning, with our trainees, that success depends on much more than honed clinical skills. Attention to detail (e.g. precision with phone numbers and email invitations) and a demeanour of grace when connections fail are essential practices. Partnering has moved beyond traditional patient/family-clinician/learner relationships.

We are fortunate to have strong collaborative relationships between our divisions (Child Psychiatry and Developmental Pediatrics), within our teams and with our trainees. We regularly reflect on and share experiences and challenges. Together, we are determined to forge and foster our partnerships during this unprecedented era of physical distance and virtual care. The key is *we* are learning *together*. As we continue to find ourselves in new spaces each day, we must continue this shared learning in spite and because of the change and uncertainty that lies ahead.

